# Biotechnological approach for improvement of *Crambe* species as valuable oilseed plants for industrial purposes

**DOI:** 10.1039/d2ra00422d

**Published:** 2022-03-02

**Authors:** Nadia Pushkarova, Alla Yemets

**Affiliations:** Institute of Food Biotechnology and Genomics, National Academy of Sciences of Ukraine Osypovskogo Str., 2a Kyiv 04123 Ukraine pushkarovano@gmail.com yemets.alla@nas.gov.ua

## Abstract

Boosting technological innovation for a sustainable and circular bioeconomy encompasses the use of renewable materials and development of highly effective biotechnological approaches to improve the quality of oilseed crops and facilitate their industrial deployment. The interest in cultivating *Crambe* as a potential crop is steadily growing due to its low propensity to crossbreeding with other oilseed crops, valuable seed oil composition and a high yield capacity. The main focus is located on *Crambe abyssinica* as the most adapted into the agriculture and well-studied *Crambe* species. At the same time, the *Crambe* genus is one of the most numerous of the *Brassicaceae* family featuring several underestimated (orphaned) species with useful traits (abiotic stress tolerance, wide range of practical applications). This review features progress in the biotechnological improvement of well-adapted and wild *Crambe* species starting with aseptic culture establishment and plant propagation *in vitro* reinforced with the use of genetic engineering and breeding techniques. The aim of the paper is to highlight and review the existing biotechnological methods of both underestimated and well-adapted *Crambe* species improvment, including the establishment of aseptic culture, *in vitro* cultivation, plant regeneration and genetic transformation to modify seed oil content and morphological traits of valuable species.

## Abstract 2

The *Crambe* species has great potential for biofuel production and a wide range of possible applications. Biotechnological methods could become a useful tool for both underestimated and well-adapted *Crambe* species improvement.

## Graphical abstract

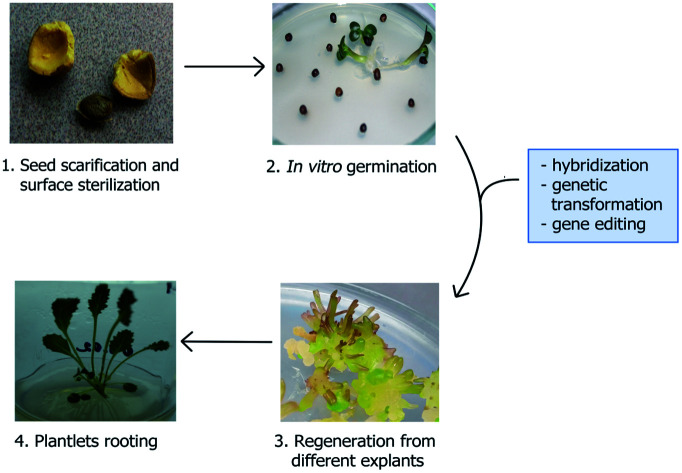

## 1. Introduction

Based on production and circulation of energy, the sustainable bioeconomy is strengthening its position – the biotechnology industry's economy was worth $62,5 billion in 2019 compared to $44,47 billion in 2017.^1,2^ New policies are being elaborated to reduce carbon emissions, improve resource efficiency, explore renewable energies and develop sustainable agriculture.^3,4^ The large-scale application of a bioeconomy envisages the use of renewable materials and highly effective biotechnologies to improve the quality of oilseed crops and their industrial implementation.^1^ Oilseed plants can be used for food or industrial purposes depending on the oil composition and it is essential to prevent the overlapping of crops to maintain high seed oil value. This course is highlighted by the European Commission in European Climate Law and aims to reduce biofuel production from food or feed crops to none by the 2030.^4^

The interest in oilseed crops has grown significantly over the last decade driven not only by spreading the application of the concept of sustainable bioeconomy but also thanks to the growing overall demand due to increasing energy consumption and limited petroleum reserves.^5^ The industry is primarily interested in the fatty acid composition of the seed oil, specifically in those with the chain length between 12 and 22 carbon atoms: palmitic (16 : 0), stearic (18 : 0), oleic (18 : 1 Δ_9_), linoleic (18 : 2 Δ_9,12_), α-linolenic (18 : 3 Δ_9,12,15_), lauric (12 : 0) and erucic (22 : 1 Δ_13_) acids. High content of the latter is of particular interest for biofuel production.^6,7^

Global challenges, such as climate change, deteriorating water and soil conditions, and global population growth are raising a challenge to improve the efficiency of food production. A promising approach to solving this problem is the use of wild, underestimated, or neglected (orphaned) crops in agriculture due to their high nutritional value, high adaptability, and resistance to stress.^8–10^ Wild *Crambe* plants are considered as one of the underutilized vegetables and an alternative crop to enhance productivity of agriculture in the abiotic stress regions.^11,12^ Therefore, it is important to estimate the possibility of wild *Crambe* species application in agriculture for food, feed and oil production either by cultivation or as a source of valuable traits for improving crops. Although there are several articles highlighting the agronomic and agricultural practices of *Crambe*,^14,15^ there are no generalized data on the use of different *Crambe* species, including wild species, to create *in vitro* tissue cultures, micropropagation and plant genetic modifications that are very important for further biotechnological improvement of valuable oilseed plants. The review is summarizing the existing biotechnological approaches for improvement of both underestimated and well-adapted *Crambe* species, and reveals the prospects for their further use in crop biotechnology.

## 2. *Crambe* plants: agronomic features and seed oil content

The *Crambe* genus is the most diverse in the *Brassicaceae* family, and consists of 44 known species.^14,16^*Crambe* species are widely spread and can grow both at the sea level and at 3800 m above the sea level in Himalayas. They can be found in marine or semi-arid and even dry climatic conditions. According to the geographical distribution this genus can be divided into three groups:^17,18^

•*Dendrocrambe* DC. – endemic species of the Macaronesian archipelago, northern part of Central Europe;

•*Leptocrambe* DC. – species of the Mediterranean and Africa;

•*Sarcocrambe* DC. – species grown in Eurasia up to the Western Himalayas.

**Fig. 1:**
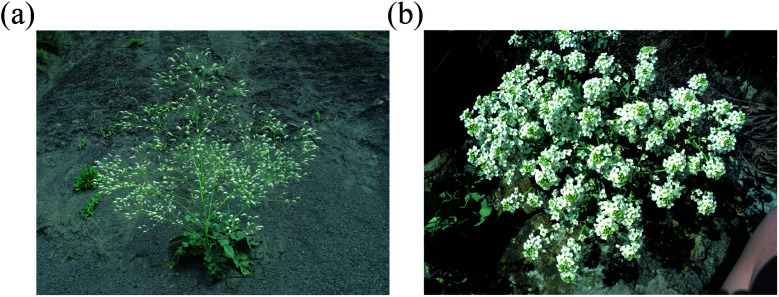
*Crambe koktebelica* (a) and *Crambe maritima* (b) plants (photo made by Kalista Maria in Karadag mountain range, Crimea, Ukraine). Bar 20 cm.

*Crambe* plants are annual and perennial grasses or subshrubs. The stem is glabrous or sparsely pubescent with simple hairs. Basal leaves are large, notched-toothed, pinnately dissected or deeply dissected, usually glabrous or sparsely pubescent, fleshy, vesicular-wrinkled and wavy at the edges on strong long petioles. Stem leaves are much smaller than the basal leaves, softer, usually toothed, petiolate and the upper leaves are small filamentous or absent. The flowers are very numerous^14,16^ and small, white or yellowish-green in color (Fig. 1). The fruit is an unopened pod. Seeds are 1–3 mm in diameter, brown, greyish-brown or black. Flowering occurs in June.^14,18^

*Crambe* basic set of chromosomes is *x* = 15, plants are always polyploid.^19,20^ The diploid set of chromosomes (2*n*) varies from 30 to 150 similarly to polyploid rows of 30, 45, 60, 90, 120 and 150 chromosomes.

*Crambe* plants are well-adapted in Europe, the USA, China, Brazil and other countries. They are mainly annual plants with short life cycle of 90 days and uniform maturation, which allows mechanical harvesting. *Crambe* plants are known to tolerate soil salinity, drought and temperature changes.^7,21,22^ Although there is only one species that is being cultivated at an industrial scale – *C. abyssinica* – there are also a number of native species that can be a valuable source of the genes required for crop improvement.

*Crambe* is characterized by a high yield capacity (similar to that of spring rapeseed) and relatively high oil content in the seeds (40%).^21^ The plants naturally contain up to 60% (*C. abyssinica*) of erucic fatty acid (22 : 1 Δ_13_) which disqualifies them for food production.^23^ Unlike other widely used oilseed crops (*e.g.* rapeseed and sunflower), *Crambe* is at a low risk of outcrossing to its wild relatives, its seed morphology is distinct from other oilseed crops. These traits make it possible to grow oilseed crops for food consumption in the immediate vicinity of *Crambe* plants without the risk of harming the quality of both food and nonfood crops and outcrossing of different traits among varieties or species.^24,25^ The main source of erucic acid is high erucic acid rapeseed (HEAR).^26^ At the same time, rapeseed (canola) is cultivated for food purpose and due to seed and plants similarity the problem of mixing and cross pollination between food and nonfood rapeseed is highly possible.^26,27^ As erucic acid is harmful to human health when consumed with food and it should not enter the food chain, the *Crambe* species could be an alternative source of erucic acid that eliminate the problem of outcrossing with rapeseed.^13,26^

*Crambe* plants (mainly *C. abyssinica*) are currently cultivated at an industrial scale in USA, Canada and Europe as they are well suited for oil production.^6,27–29^ The oil from seeds is used for production of plastic films, adhesives, nylon, thermal insulation, corrosion inhibitors, synthetic rubber and industrial lubricant.^30–33^ It can also be used for biodiesel production^34^ due to its high resistance to degradation and oxidation.^35^ Refined *Crambe* oil can be used for cosmetics and waxes production.^7,24^ The waste after seed oil extraction can potentially be used for animal feed as a protein supplement due to the high content of crude protein (up to 45%).^31,36–45^

## 3. The use of underestimated wild *Crambe* species

As mentioned above, despite the fact that *Crambe* genus is known to possess valuable qualities for agriculture and industry, only one species of the genus, *Crambe abyssinica*, has been widely adapted in agriculture and cultivated mainly for its oil that is rich in erucic acid.^7,46^*Crambe* from *Leptocrambe*, *Crambe*, groups are the most numerous and all the species in *Leptocrambe* group are shown to have erucic acid at levels comparable to those of *C. abyssinica*.^47^

There are numerous *Crambe* species beside *C. abyssinica* that have been used for food, feed or other purposes.^48–51^ Out of a wide range of the species the most common ones used for food are *C. maritima* (Sea Kale), *C. cordifolia* (Giant Colewort), *C. orientalis*, *C. tataria* (Tartar Bread Plant), *C. kotschyana*,^52^*C. aspera*, *C. koktebelica*, *C. pinnatifida* and *C. steveniana*.^51^*Crambe* leaves and roots are known to have been consumed since ancient times. *C. maritima* (or Sea Kale) leaves were considered a delicacy in ancient Rome and they were also in France and England in the 19^th^ century.^53^ In the 1990s, there were attempts to introduce this plant as a vegetable on a large scale in France. Nowadays, Sea Kale is considered a sports food and a food supplement with high crude fiber and protein content.^50^ Green mass also was shown to have potent antioxidant activity and high polyphenol compounds content depending on the plant development stage^54^ and a possible application in medicine.^51^ Also, the antimicrobial activity was shown for underground parts of wild *Crambe* plants.^54^

*Crambe* plants are great for ruminant animal and fish feed as seed meal contains 45–58% of protein with well-balanced amino acid content (especially high levels of lysine and methionine).^36–45^ It could be used as high value feedstock protein, and the aerial part is excellent for hay due to a high biomass yield containing up to 50% of crude protein.^13,55,56^

Despite application in food and feed, green mass as well as seed meal of some *Crambe* species contain glucosinolates that are considered toxic for animal and human health.^57^ However, physical treatment (freezing or boiling) leads to near complete decomposition of glucosinolates thus making the consumption of sprouts and leaves safe.^58^ Therefore, reducing levels of glucosinolates or disposing of them completely could make *Crambe* seed meal suitable for ruminant animals. A potential strategy for this may include reduction of the expression levels of the key genes in the glucosinolates biosynthesis pathway.

## 4. Biotechnological approach for *Crambe* improvement

### 4.1. *Crambe in vitro* aseptic culture establishment

According to the available literature the establishment of *Crambe* aseptic culture is carried out mainly through seeds that are characterized with high germination capacity. Seed germination correlates with the seed development phase and environmental conditions.^59^

Despite high germination capacity seed dormancy was also reported for *Crambe* species.^59,60^ As recommended by Gutormson *et al.*,^61^ application of 0.2% potassium nitrate (KNO_3_) solution is a possible way for breaking seed dormancy of freshly harvested seeds (it should not be applied for seeds that have been stored for more than nine months).^62^ The other way of breaking seed dormancy is removal of pericarp which was reported by Nunes *et al.*^62^ as a minor limiting factor for *Crambe* seeds germination. However, pericarp and seed coat removal of *C. giberosa* was an efficient way to establish high seed germination and greatly accelerate this process *in vitro*.^63^ Our previously obtained results support the positive effect of pericarp and seed coat removal for successful and fast *in vitro* germination of several *Crambe* species native for Ukraine (*C. koktebelica*, *C. tataria*, *C. aspera*, *C. steveniana*. *C. maritima*, *C. grandiflora*, *C.cordifolia*, *C. mitridatis*).^64–68^ It is necessary to mention that, although pericarp removal can be easily performed at an industrial scale, it could increase the risk of seed damage and spreading of fungal infection, so it should be performed when the risk is justified.

The first step in obtaining an aseptic culture is explant surface sterilization of the plant. The result can vary depending on the explant type and sterilizing agent of choice. Seed surface sterilization is a way to obtain aseptic plant culture in the least damaging way for the plant material. The highest number of aseptic plantlets was obtained from the seeds surface sterilization with diocidum (the exposure time 2–3 minutes)^64–68^ or commercial bleach solution (exposure time 10–20 minutes).^69–71^ Also, 3% hydrogen peroxide for 10 minutes was successfully used for that purpose.^63^ The use of antibiotics for elimination of bacterial contamination was shown to be useful. Immersion in a solution containing penicillin and rifampicin (10 mg L^−1^) before the application of seed surface sterilization protocol was successful to obtain an aseptic culture.^71^ After the seed surface sterilization seeds were transferred to culture medium of choice (mainly MS or half strength MS) in culture chamber at 22–25 °C with 16-hour photoperiod.^64–71^ Time of *Crambe* aseptic seed germination after the seed surface sterilization procedure varied and lasted from 3 days to a month depending on the species, seed storage time and conditions, and on the seed coat removal.^64–73^ For aseptic culture establishment explants from aseptic seedlings were taken and transferred to a medium for further cultivation and micropropagation.

Considerable efforts have been made to establish *Crambe* green shoots surface sterilization. For that purpose, *C. gibberosa* shoots were soaked in soap solution with Tween80 for 10–15 minutes. Then, the shoots were washed in distilled water, soaked in 0.1% HgCl_2_ solution for 8 minutes and cut into small nodal explants. Cut ends were paraffined to prevent direct impact of sterilization solution.^63^ After the surface sterilization, the nodal explants were transferred to MS medium^74^ containing 6-benzylaminopurine (BA) 2.5 mg L^−1^ in culture chamber at 23–25 °C for 16 hour long photoperiod. After 6–7 days of the cultivation, the nodal explants turned brown and no regeneration was observed.^63^

### 4.2. *Crambe* micropropagation

Further aseptic plantlets micropropagation is performed mainly on the MS solid medium although the choice of nitrogen and carbon source can influence regeneration of *Crambe in vitro*. Nitrogen is essential for plants growth and development. Aseptic plantlets uptake nitrogen mainly in the form of NO_3_^−^ and NH_4_^+^ ions therefore its sufficient availability in both forms is important for cell growth and differentiation and the balance of these two nitrogen compounds is needed. Lepoivre,^75^ MS^64–71^ or N6 (ref. 75) media have been reported as the most efficient nutrient solutions with optimal mineral composition for *Crambe* regeneration.^75^

The choice of gelling agents for the medium is also important for successful regeneration *in vitro*. Gelling of the regeneration medium with agar leads to callogenesis and direct somatic embryogenesis and the use of phytobland contributes to indirect shoots regeneration.^73^ For higher *Crambe* regeneration *in vitro* AgNO_3_ is also used. It has been found that addition of 5 mg L^−1^ AgNO_3_ into the medium improves plantlet regeneration frequency.^73,75^

Depending on the type of explant and growth regulators content in the medium the plant regeneration frequency varies for each species therefore genetic variations of the plants should be taken into account. Efficient *in vitro* plant regeneration protocols have been obtained only for several *Crambe* species – *C. abyssinica*,^73^*C. giberosa*,^63^*C. orientalis*,^70^*C. tataria*,^76^*C. maritima*.^77^ Propagation of *Crambe* plants *in vitro* was mainly carried out according to the protocols – by direct shoot regeneration of differentiated tissues from different explants:^63,70,75–77^

•Cotyledons (*C. giberosa* and *C. abyssinica*, *C. orientalis*);

•Hypocotyls (*C. abyssinica*, *C. orientalis*);

•Lateral buds (*C. giberosa*, *C. abyssinica*);

•Apical meristems (*C. abyssinica*);

•Leaf parts (*C. giberosa*, *C. tataria*, *C. abyssinica*);

•Root parts (*C. maritima*, *C. tataria*, *C. giberosa*);

•Stem parts (*C. abyssinica*).

Morphogenic potential of leaf explants was estimated for *C. giberosa*, *C. tataria* and *C. abyssinica* but only the ability for callus formation was observed.^63,73,76^ For *C. abyssinica* petiole explants indirect somatic embryogenesis frequency was up to 2.5% while for stem explants it was up to 30%.^73^

Organogenesis from *C. giberosa*, *C. maritima* and *C. tataria* root explants has been studied.^63,76,77^ Growth regulators were found to stimulate the morphogenic potential. Growing root explants on MS medium supplemented with a combination of cytokinins and auxins contributed to somatic embryogenesis for *C. giberosa* (0.5 mg L^−1^ of BA),^63^ for *C. maritima* (2.0 mg L^−1^ of IAA and 0.8 mg L^−1^ of kinetin),^77^ and for *C. tataria* (NAA 1–2 mg L^−1^ and BA 1–2 mg L^−1^).^76^

The use of root explants for plant propagation of *C. giberosa*, *C. maritima* and *C. tataria* in aseptic culture proved to be quite effective provided that the appropriate amount of NAA and BA was added to the medium.

Intensive callogenesis was also shown for *C. tataria* leaf explants. It was noted^76^ that both cytokinins and auxins were essential for callus tissue formation (1 mg L^−1^ 2,4-D induced callogenesis on leaf explants), while NAA was less effective than 2,4-D, and BAP, in turn, was less effective than kinetin. Thus, the highest intensity of callus tissue growth on leaf explants was observed on medium with 2 mg L^−1^ of kinetin and 1 mg L^−1^ of 2,4-D. *C. tataria* also showed a low callogenesis activity of root explants, compared to leaf explants. For root explants, the highest activity of callogenesis was observed with the use of medium supplemented with 2 mg L^−1^ of 2,4-D.^76^

### 4.3. *In vitro* plant regeneration of wild *Crambe* species

Morphogenic potential of different types of explants of several *Crambe* species was previously tested by us (Table 1). This research was performed to estimate the possible use of root, leaf and petiole explants for *in vitro* propagation of *Crambe* plants that are endangered in Ukraine, as well as in other counties, and to obtain the basis for *Crambe* gene pool improvement.^64–68,78^

**Table 1:** Recommended growth regulators content in the medium for *in vitro* plant regeneration from different types of explants of *Crambe* species

Species	Lateral bud explants	Leaf explants	Petiole explants	Root explants	References
*C. koktebelica*	BA 1 mg L^−1^	BA 5 mg L^−1^ + NAA 0.5 mg L^−1^	BA 2.5 mg L^−1^ + NAA 0.1 mg L^−1^	BA 1 mg L^−1^ + NAA 0.1 mg L^−1^	64, 67, and 78
*C. tataria*	BA 0.3 mg L^−1^	BA 1 mg L^−1^ + NAA 0.1 mg L^−1^	BA 1 mg L^−1^ + NAA 0.1 mg L^−1^	Kinetin 1 mg L^−1^ + NAA 0.5 mg L^−1^	65, 76, and 78
*C. aspera*	Kinetin 0.5 mg L^−1^	BA 5 mg L^−1^ + NAA 0.5 mg L^−1^	BA 5 mg L^−1^ + NAA 1 mg L^−1^	BA 1 mg L^−1^ + NAA 0.1 mg L^−1^	68 and 78
*C. steveniana*	BA 0.6 mg L^−1^	BA 1 mg L^−1^ + NAA 0.1 mg L^−1^	BA 2.5 mg L^−1^ + NAA 0.1 mg L^−1^	Kinetin 1 mg L^−1^ + NAA 0.1 mg L^−1^	78
*C. maritima*	BA 1 mg L^−1^	BA 2.5 mg L^−1^ + NAA 0.5 mg L^−1^	BA 2.5 mg L^−1^ + NAA 0.1 mg L^−1^	BA 1 mg L^−1^ + NAA 0.1 mg L^−1^	78

**Fig. 2:**
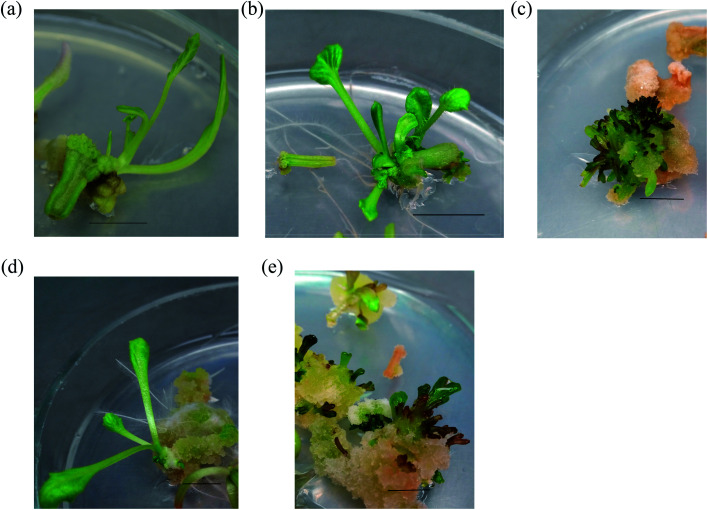
Plantlets regeneration on petiole explants: (a) *C. koktebelica*; (b) *C. tataria*; (c) *C. aspera*; (d) *C. steveniana*; (e) *C. maritima*. Bar 1 cm.

Morphogenic potential of root explants of wild *Crambe* species has been researched and showed rather low regeneration frequency for four species (Fig. 2): *C. koktebelica* – 20% (BA 1 mg L^−1^ and NAA 0.1 mg L^−1^), *C. aspera* – 10% (BA 1 mg L^−1^ and NAA 0.1 mg L^−1^), *C. tataria* – 30% (Kinetin 1 mg L^−1^ and NAA 0.5 mg L^−1^) and *C. steveniana* – 40% (Kinetin 1 mg L^−1^ and NAA 0.1 mg L^−1^) (Table 1). The highest micropropagation rates were observed in the case of cultivation with low cytokinin and auxin content in the medium. Plant regeneration from *C. koktebelica* and *C. aspera* roots took place only on the medium with BA. *C. maritima* root explants had the highest micropropagation rates on the medium with either BA or kinetin, all of its explants formed plantlets on the MS medium with 1 mg L^−1^ of BA and 0.1 mg L^−1^ of NAA.

The conducted experiments have demonstrated that regeneration frequency of leaf explants was higher than that of root explants. New plantlets for *C. koktebelica*, *C. tataria*, *C. aspera*, *C. steveniana* and *C. maritima* were formed on the medium containing either BA and NAA, or kinetin and NAA, however, the former combination (BA and NAA) showed the highest propagation rates. The relation between the regeneration frequency and auxin content in the medium was also established, lower NAA concentration (0.1–0.5 mg L^−1^) made for higher propagation rates. Thus, the highest regeneration frequency from leaf explants was as follows: 28% for *C. koktebelica* (BA 5 mg L^−1^ and NAA 0.5 mg L^−1^), 38% for *C. tataria* (BA 1 mg L^−1^ and NAA 0.1 mg L^−1^), 50% for *C. aspera* (BA 5 mg L^−1^ and NAA 0.5 mg L^−1^), 80% for *C. steveniana* (BA 1 mg L^−1^ and NAA 0.1 mg L^−1^) and 100% for *C. maritima* (BA 2.5 mg L^−1^ and NAA 0.5 mg L^−1^) (Table 1).

We have also established that NAA concentration in the medium was an important regulatory factor for both petiole and leaf explants regeneration rate, the concentration 0.1–0.5 mg L^−1^ of NAA resulted in the higher propagation rates. Plantlets from petioles were formed on the medium with either BA or kinetin (combined with NAA). 100% regeneration frequency occurred for *C. tataria* (BA 1 mg L^−1^ and 0.1 mg L^−1^), *C. aspera* (BA 5 mg L^−1^ and 1 mg L^−1^), *C. steveniana* (BA 2.5 mg L^−1^ and NAA 0.1 mg L^−1^) and for *C. maritima* (BA 2.5 mg L^−1^ and NAA 0.1 mg L^−1^). The highest propagation rates for *C. koktebelica* petiole explants (60%) were noted on the medium with 2.5–5 mg L^−1^ of BA and 0.1 mg L^−1^ of NAA (Table 1).^64–68,78^

**Fig. 3:**
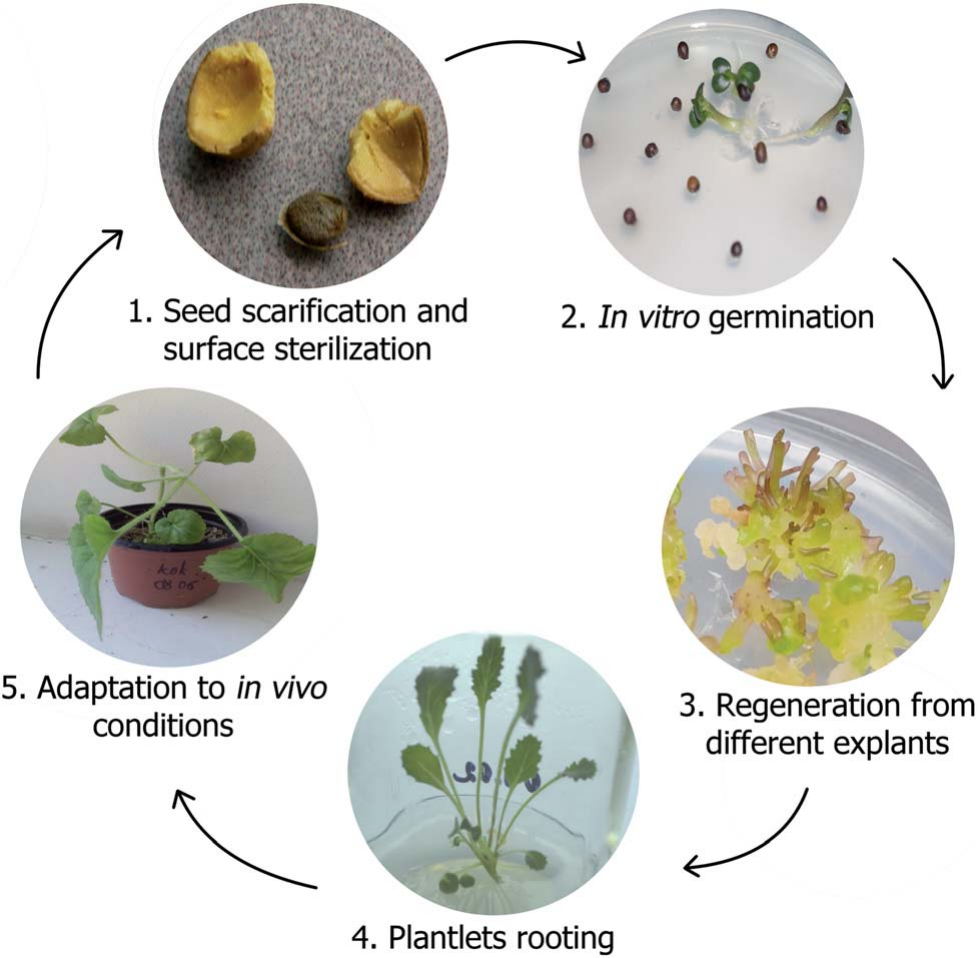
*C. aspera in vitro* micropropagation and plant adaptation to greenhouse conditions.

**Fig. 4:**
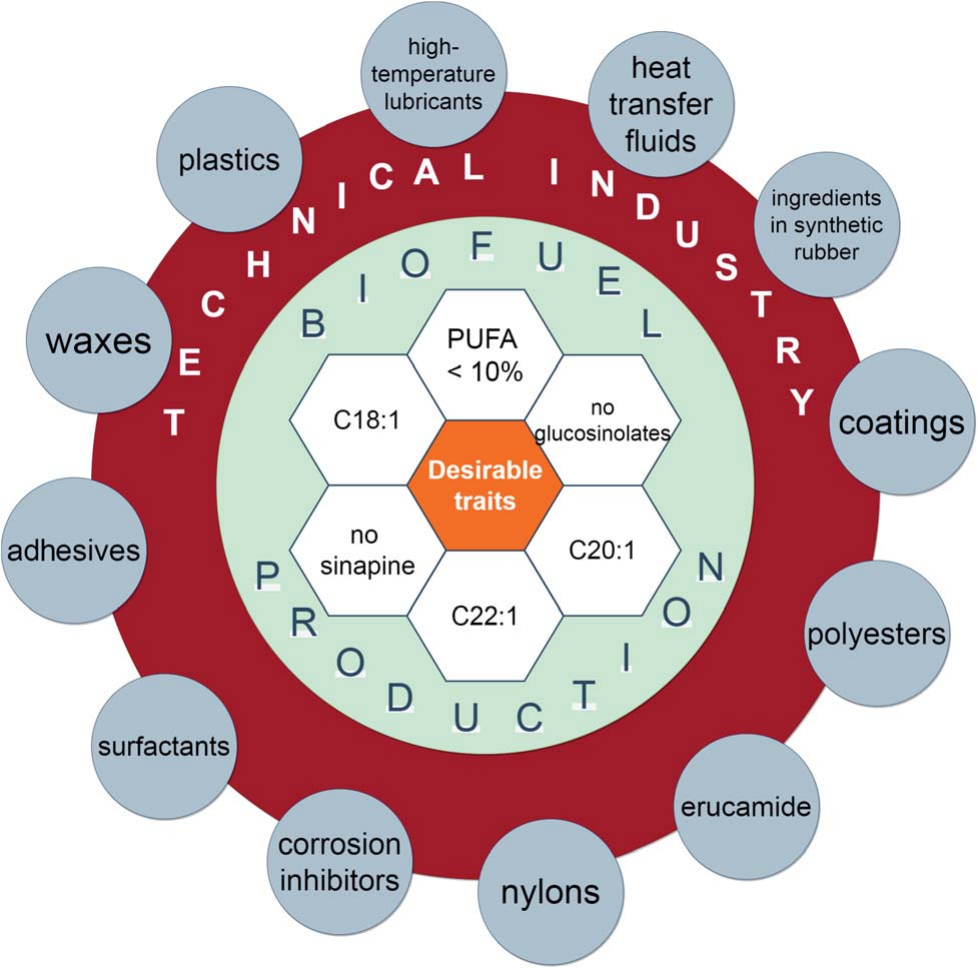
The various components of *Crambe* species for possible applications in biofuel production and technical industry.

Our previously conducted research provides a full way from a *Crambe* seed to numerous plants in greenhouse *via in vitro* propagation with rooting and acclimatization of plants for several species (Fig. 3). It can therefore be inferred, that a platform for further biotechnological improvement of *Crambe* species is established and is applicable to other relevant methods. Underestimated *Crambe* species are often endemics and endangered^14,79,80^ therefore the problem with reproduction in their natural habitats or law restrictions due to the threatened status of plants is present. *In vitro* propagation could provide with rapid multiplication of plants that have characteristics of mother plant without considerable damage to the population in their habitats. *Crambe* genetic breading for increasing oil and other compounds production requires high efficiency of biotechnological steps.^75^ Establishment of propagation protocols for each of *Crambe* species is fairly important. Propagation protocols can be further used as a platform to transfer desirable traits (Fig. 4) *via* cellular and genetic engineering approaches or to generate new lines with desirable traits due to somaclonal variability that can occur by chance when plant tissues are cultured *in vitro*.^81^ By combining existing propagation protocols for underestimated species with the knowledge on genetic constructs used for *C. abyssinica* for enhancing desirable traits the new varieties could be obtained.

### 4.4. Somatic hybridization as a method for *Crambe* improvement

Somatic hybridization is a method of cellular genome manipulation by protoplasts fusion of two different species to form a new hybrid plant with combined features. It may be intraspecific, interspecific, intrageneric and intergeneric. Somatic hybridization can be performed between different varieties of one species and between different species even not closely related. As a result, the hybrids similar to polyploid plants can be obtained with the chromosomes of both plants. Plant protoplasts fusion as a method of somatic hybridization is an essential approach to overcome sexual incompatibility between different plant species thus providing a tool for new genetic traits formation. The most common recipients for *Crambe* genome transfer *via* somatic hybridization are *Brassica* spp., namely *B. napus*, *B. campestris*, *B. juncea*.^82,83^ Major limitations were found in the intergeneric cross process between *Brassica* spp. and *C. abyssinica* such as prefertilization incompatibility and embryo abortion at multi-cellular globular stage.^82^ To overcome an embryo abortion ovary culture was successfully used and *B. juncea* × *C. abyssinica* hybrids were obtained.^80^ Wang *et al.*^82^ demonstrated a successful transfer of new allelic variants of the FAE1 (fatty acid elongation gene controlling erucic acid biosynthesis) from *C. abyssinica* into *B. napus via* somatic hybridization. Overexpression of the *C. abyssinica FAE1* gene in *B. carinata* resulted in a substantial increase in the content of erucic acid in seeds compared to the wild type control.^84^

UV-irradiated (0.10 J cm^−3^ dose) protoplasts from *C. abyssinica* leaves were mixed *in vitro* with *B. napus* protoplasts (in 1.2 : 1 ratio) and treated with 40% polyethylene glycol, then cultivated in the dark for further microcalli formation and hybrid plant regeneration. Obtained asymmetric somatic hybrids had from 2 to 40 more chromosomes than expected for *B. napus* (38 chromosomes) and showed the presence of some characteristic bands from *C. abyssinica* (confirmed by the amplified fragment-length polymorphism analysis (AFLP)). The obtained hybrids had high pollen viability, could be fertilized and set seeds. The subsequent seed oil content estimation showed several asymmetric somatic hybrids with an increased erucic acid content and seed set.^17^

Intraspecific hybridization as a way of transferring desirable traits of related wild species (diseases resistance, abiotic stress resistance, higher yield *etc.*) to well-adapted and cultured crop *C. abyssinica*^85–88^ was used by Du *et al.*^89^ Though, this approach is limited due to the sexual incompatibility between species that leads to low fertility of hybrids or the abortion of embryo at early development stages, such obstacles can be overcome by protoplast fusion and embryo rescue.^90,91^ Hybrids of *C. abyssinica*, *C. hispanica* and *C. kralikii* can be obtained with or without embryo rescue.^89^

### 4.5. *Agrobacterium*-mediated genetic transformation of *Crambe*

*Agrobacterium*-mediated transformation is a widely used method of plants genetic engineering due to its high efficiency, which is influenced by several factors such as plant donor health, stage of the donor material, vector type, regeneration and selection conditions efficiency.^23,72,92,92^ As *Crambe* plants are considered highly potential oilseeds and a source of desirable genes for crop improvement, its genetic transformation is aimed to change the seed fatty acid composition and increase the very long chain fatty acids (VLCFA) content in seeds.^84,94–96^

Genetic modification of *Crambe* plants became possible after the development of the first successful plant regeneration and the transformation protocol mediated by *Agrobacterium*.^93^ To overcome the so-called bottleneck in erucic acid accumulation^97^ in attempt to decrease polyunsaturated fatty acids content in *Crambe* seeds, up to four gene-combined constructs were created and used for transformation.^96^ The first attempt to apply gene stacking strategy for increasing erucic acid content in *Crambe* was performed by Li *et al.*^23,72^ For this purpose, they used vectors pHAN, pWatergate and three-gene construct harbouring the *LdLPAAT* (lysophosphatidate acyltransferase gene from *Limnanthes douglasii*),^98^*CaFAD2-RNAi* (fatty acid desaturase 2 gene) and *BnFAE1* (fatty acid elongase 1 gene from *B. napus*) genes driven under the napin promoter and with *nptII* selectable marker gene^23^ and binary vector pCAMBIA carrying *BnFAE1* and *LdLPAAT* with *hpt* selectable marker gene.^72^ It has been shown that incorporation of *FAD2-RNAi* together with the previously mentioned genes resulted in the increase of erucic acid amount in *Crambe* seed oil up to 70%.^23,72,94^

*Crambe* gene could be a useful source for improving oilseed crops by cross-species silencing.^99^ RNAi-silencing construct containing *Crambe FAD2, FAD3* and *FAE1* genes was used for silencing the genes of related *Arabidopsis thaliana* resulting in fatty acid content alteration. Expression of *CaFAD2-FAE1* gene silencing constructs decreased *cis*-11 eicosenoic (20 : 1) and linoleic (18 : 2 Δ_9,12_) and CaFAD3-FAE1 decreased α-linolenic (18 : 3 Δ_9,12,15_) fatty acid content in seed oil.^99^ The same constructs were used for developing ultrahigh oleic oil content into *C. abyssinica* resulting in a significant increase in oleic acid (18 : 1 Δ_9_) content wherein, and this trait was stable during several generations.^96^ Suppressing of the lysophosphatidic acid acyltransferase LPAAT2 expression was also performed *via* RNAi targeting with *CaFAD2* and *CaLPAAT2* genes from *Crambe*.^26^

*Crambe* seed oil contains almost 70% of VLCFAs which make it an attractive source for the biotechnological production of industrial oils by overlaying the wax ester biosynthetic pathway from jojoba onto the existing triacylglycerol biosynthetic pathways of *C. abyssinica*. Wax ester biosynthesis requires the fatty acid esterification from an acyl-CoA substrate to a fatty alcohol, bypassing the fatty acid incorporation onto glycerol backbones to form triacylglycerols. For this purpose, *ScFAR* and *ScWS* cDNAs were co-expressed under control of strong seed-specific promoters in *Crambe* resulting in successful tailoring wax ester profiles.^24^ Despite promising results in altering FA content for wax esters, a production field and greenhouse trials showed that transgenic lines expressing *ScWS* and *ScFAR* genes provided normal growth of transgenic plants but with a slightly reduced seed yield, oil content and germination rate compared to the wild type with delayed flowering and fruit set. *Crambe* lines selected for a field testing had approximately 25% of the oil as wax esters, with the remainder in the form of TAG.^24,100^

### 4.6. *Crambe* hairy roots culture establishment

Genetic transformation by means of *Agrobacterium rhizogenes* results in hairy roots culture formation that is caused by root loci genes incorporation into the plant DNA. Hairy root culture is characterized by rapid hormone-independent growth, lateral branching, high genetic and biochemical stability and can produce valuable secondary metabolites non inherent for the initial plant.^101,102^ This technology is used for secondary metabolites production, plants biochemical properties study and could be used at an industrial scale in bioreactors.^103^ The possibility to use hairy root culture for oil production in bioreactors was also reported.^104,105^

*Crambe* hairy root culture was established using leaf and cotyledons explants inoculated with *A. rhizogenes* A4 and 15 834 strains by two methods, needle inoculation and sonication.^106^ It should be noted that addition of 200 mM acetosyringone (4-acetyl-2,6-dimethoxyphenol) to the growth medium, used for *A. rhizogenes* and inoculated plants explants growth, increased transformation efficiency. Hairy root culture that was incubated for more than three weeks showed symptoms of aging (lack of growth and culture browning due to intensive phenolic compounds excretion) but admixing polyvinylpyrrolidone reduced the culture aging and intensified its development.^106^ The hairy root culture establishment was studied with the use of cotyledons and leaf explants and it was shown that the leaf explants were of better choice for *Crambe* transformation resulting in 16% transformation efficiency.^106^ This work^106^ was the first attempt to obtain stable *C. abyssinica* hairy roots culture that could be characterized by genetic stability, rapid growth on hormone-free medium and possible high polyunsaturated fatty acids production. These traits make it possible to use hairy roots culture in bioreactors for oil production. For this purpose, hairy roots fatty acids content of different *Crambe* species should be studied further.

Fatty acids content in the hairy root culture was similar to that of non-transformed roots but differed only in terms of quantity. Dominating fatty acids of *A. rhizogenes*-induced roots were α-linolenic (18 : 3 Δ_9,12,15_), palmitic (16 : 0), and linoleic (18 : 2 Δ_9,12_) acids. Diacylglycerols, free fatty acids, triacylglycerols, and sterol esters, found in root culture and polar lipids, were the dominant class of lipids.^105^ The obtained results provide the protocol of hairy root culture establishment and reveal some features of anabolism and catabolism of *Crambe* root lipids.

Further attempts of improving *Crambe* hairy roots lipid metabolism were focused on introducing genes coding for the fatty acyl-CoA reductases from *A. thaliana* (*AtFAR5* gene) and *Simmondsia chinensis* (*ScFAR* gene). *A. rhizogenes* A4 and ATCC 15834 stains carrying binary plasmids pBIN-AtFAR5, pGW-AtFAR5, pBIN-ScFAR and pGW-ScFAR were applied for the transformation of *C. abyssinica* leaf explants.^107^ The reported transformation efficiency was 35–45% and the obtained hairy roots assessment showed different fatty alcohol profiles – culture containing *AtFAR5* gene was unable to produce fatty alcohols but for the lines carrying *ScFAR* gene stearyl alcohol (18 : 0-OH), arachidyl alcohol (20 : 0-OH) and behenyl alcohol (22 : 0-OH) production was noted.^107^

Hairy root culture is a valuable source for fatty acids production but further research should be conducted to estimate the full potential of this system and to see if it could compete with the conventional seed oil production.

### 4.7. The potential of CRISPR/Cas9 gene editing for improvement of *Crambe* seed oil content and other traits

Due to a continuously growing demand for food and energy new ways to increase production of vegetable renewable oil are required. Genome editing techniques, such as clustered regularly interspaced short palindromic repeats-associated protein (CRISPR/Cas9), have emerged as a powerful, highly specific and eco-friendly tool for crops improvement.^108,109^ This system comprises Cas nuclease which makes double strand DNA breaks and a small non-coding single-guide RNA that leads Cas to the destinated genomic locus (DBS) are then repaired by plant inherent cell repair mechanisms.^110^

*Crambe* plants are a dedicated source of fatty acids that are accumulated in the form of triacylglycerols in seeds.^111^ Triacylglycerol formation is a complex process that could be altered at certain steps to overcome the bottleneck in erucic acid accumulation^97^ and change polyunsaturated fatty acid content in *Crambe* seed oil. Considerable efforts have been made for oilseed crops gene editing by means of CRISPR/Cas9 with targeting genes that are involved in triacylglycerol synthesis: lysophosphatidic acid acyltransferase,^112^ fatty acid desaturase,^113–115^ fatty acid elongase,^116^ diacylglycerol acyltransferase.^117^ At the same time, there is no data available on *Crambe* gene editing *via* CRISPR/Cas9 system, therefore the study of this system application to *Crambe* is of particular interest.

## 5. Conclusions

*Crambe* plants have great potential and a wide range of technological application. The considerable efforts have been made to produce biotechnological systems for *Crambe* improvement starting from *in vitro* regeneration protocols and somatic hybridization to *Agrobacterium*-mediated genetic transformation with genes regulating lipid production. Further research in this field could provide a useful genetic material for crop improvement as well as new systems for producing valuable fatty acids.

## Author contributions

Nadia Pushkarova carried out the literature search, summarized data and wrote the paper. Alla Yemets reviewed and edited the manuscript, analyzed the manuscript contents and made the manuscript corrections. All authors have read and approved the final manuscript version.

## Conflicts of interest

There are no conflicts to declare.

## Supplementary Material
